# Disparities in Outcomes following Resection of Locally Advanced Rectal Cancer

**DOI:** 10.3390/curroncol31070280

**Published:** 2024-06-30

**Authors:** William Y. Luo, Dimitrios N. Varvoglis, Chris B. Agala, Lydia H. Comer, Pragna Shetty, Trevor Wood, Muneera R. Kapadia, Jonathan M. Stem, José G. Guillem

**Affiliations:** Division of Gastrointestinal Surgery, Department of Surgery, University of North Carolina, Chapel Hill, NC 27599-7081, USAdvarvo@ad.unc.edu (D.N.V.); chris_agala@med.unc.edu (C.B.A.); jonathan_stem@med.unc.edu (J.M.S.)

**Keywords:** rectal cancer, NCDB, disparities, margins, CRM, facility type

## Abstract

Surgical margins following rectal cancer resection impact oncologic outcomes. We examined the relationship between margin status and race, ethnicity, region of care, and facility type. Patients undergoing resection of a stage II–III locally advanced rectal cancer (LARC) between 2004 and 2018 were identified through the National Cancer Database. Inverse probability of treatment weighting (IPTW) was performed, with margin positivity rate as the outcome of interest, and race/ethnicity and region of care as the predictors of interest. In total, 58,389 patients were included. After IPTW adjustment, non-Hispanic Black (NHB) patients were 12% (*p* = 0.029) more likely to have margin positivity than non-Hispanic White (NHW) patients. Patients in the northeast were 9% less likely to have margin positivity compared to those in the south. In the west, NHB patients were more likely to have positive margins than NHW patients. Care in academic/research centers was associated with lower likelihood of positive margins compared to community centers. Within academic/research centers, NHB patients were more likely to have positive margins than non-Hispanic Other patients. Our results suggest that disparity in surgical management of LARC in NHB patients exists across regions of the country and facility types. Further research aimed at identifying drivers of this disparity is warranted.

## 1. Introduction

Despite a reduction in colorectal cancer (CRC) morbidity and mortality in the general population, disparities persist among racial and ethnic groups [1,2,3,4,5]. Since a significant fraction (approximately 46,000 new cases in 2023) of the CRC burden is attributed to rectal cancer, a disease entity which requires a multidisciplinary approach, ample opportunities exist across the continuum of multimodality rectal cancer therapy for reducing disparity in rectal cancer care [6]. Although the current multimodality management of locally advanced rectal cancer (LARC) (T_3–4_, and/or N_+_, stage II and III) includes radiation and chemotherapy, most recently referred to as total neoadjuvant therapy (TNT), surgery remains an essential component, as a negative (R0) resection margin is required in order to obtain optimal cancer-specific outcomes [7,8]. However, despite advances in surgical techniques and multimodality therapy, rates of positive margins, such as the circumferential resection margin (CRM), which is defined as the closest distance of the tumor to the mesorectal fascia, remain high in the United States [9,10].

The CRM is a crucial prognostic factor impacting local and distant recurrence as well as disease-free survival, especially following preoperative combined modality therapy [10,11,12,13]. Factors associated with risk of obtaining a positive CRM following resection of a LARC include surgical technique, body habitus (obesity, narrow, deep male pelvis), emergency surgery, incomplete oncological preoperative staging, and lack of preoperative chemoradiation therapy utilization, when indicated [3,8,10,11,14,15]. Although previous studies have demonstrated an increased likelihood of positive CRM in non-Hispanic Black (NHB) compared to NHW patients, these studies did not specifically focus on LARC [16,17], nor adequately control for the type of operation performed or the administration of preoperative radiotherapy [17]. In order to further understand the factors driving the increased likelihood of positive CRM in NHB patients, our study focuses on LARC and rigorously controls for multiple potential covariates, such as the type of surgery and the administration of radiotherapy, and examines whether this disparity persists across different regions of the country and within different facility types.

## 2. Methods

The hospital-based American College of Surgeons (ACS) National Cancer Database (NCDB) was queried to identify patients diagnosed with LARC (i.e., stage II and III) who underwent surgical resection from 2004 to 2019. Patients were excluded if they (1) were under 18 years; (2) were not surgically treated or received local excision only; (3) had an unknown sequence of radiation versus surgery; and (4) had unknown pathologic grade. Patients were then stratified into the following four groups based on race and ethnicity: (1) NHW; (2) NHB; (3) Hispanic; and (4) non-Hispanic Other (NHO). The primary outcome of interest, margin positivity, was assessed as a binary outcome (Yes/No). The primary predictors of interest were race and ethnicity. Secondary predictors of interest included (1) United States (US) region and (2) facility type. Wilcoxon rank sum test and chi square tests were used to compare mean age across groups and categorical variables, respectively. To make the categories of race and ethnicity more comparable and reduce potential confounding and bias, an inverse probability of treatment weight (IPTW) [18] was generated using a multinomial logistic regression model where the outcomes were race and ethnicity. Predictors included the following confounders: age, sex, Charlson–Deyo score [19], pathologic stage, days from diagnosis to surgery, pathologic grade, type of surgery, timing of radiation therapy in relation to surgery (defined as either (1) no radiation, (2) intraoperative radiation, (3) neoadjuvant radiation, (4) adjuvant radiation, or (5) both neo- and adjuvant radiation), insurance type, high school education, and direct distance from patient’s residence to hospital. Overall mean weight was used to assure between-group balance. Once between-group balance was ascertained, binary logistic regression was used to generate odds ratios (OR), 95% confidence intervals (CI), and p-values to assess associations between margin positivity and race and ethnicity groups, and adjusted for the effect using the IPTW weight, region of the United States, defined as (1) northeast, (2) midwest, (3) south, or (4) west, and facility type, defined as (1) academic/research, (2) network cancer program, or (3) community cancer program. Supplement A lists the breakdown of states assigned to each region of the USA. Finally, interaction terms in logistic regression models were used to assess differences in margin positivity within and across regions and facility types with respect to race/ethnicity.

All tests were two-sided at alpha = 0.05. R version 4.2.2 (Comprehensive R Archive Network, Vienna, Austria) and R Studio Version 2022.12.0+353 (Posit Software, PBC, Boston, MA, USA) were used for data management. All analyses were conducted using SAS version 9.4 (SAS Inc., Cary, NC, USA).

## 3. Results

### 3.1. Baseline Demographics

**Figure 1:**
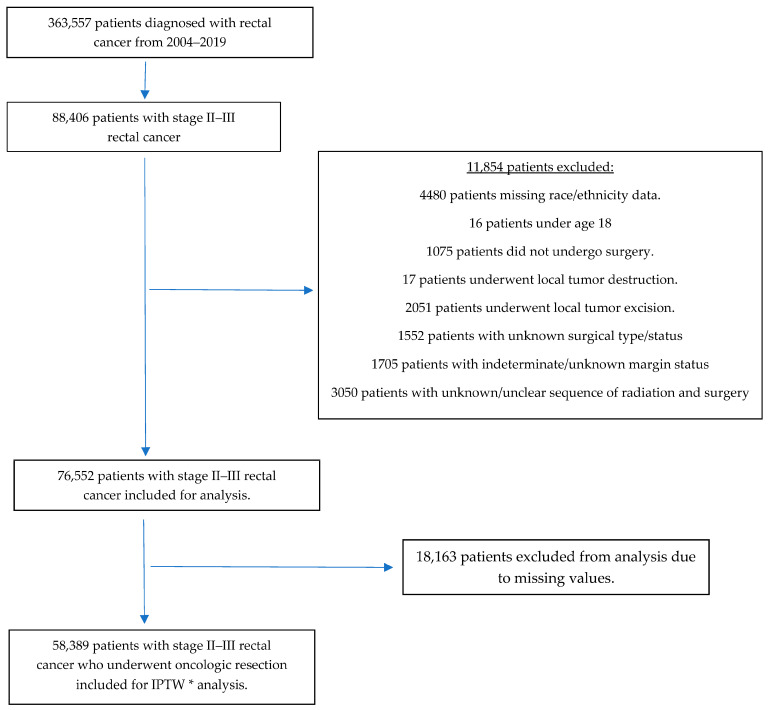
Inclusion and exclusion flow chart * Inverse-probability treatment weighting.

Our initial query of the NCDB identified 363,557 patients diagnosed with rectal cancer between 2004 and 2019, of whom 88,406 had pathologic stage II and III and were included in the analysis. After excluding patients with missing data for our confounders of interest, 58,389 patients remained and underwent analysis. Figure 1 summarizes our inclusion and exclusion process.

Of the included patients, 81%, 8%, 6%, and 5% were NHW, NHB, Hispanic, and NHO, respectively, 60% were male, and the mean age was 62 years. Table 1 summarizes additional baseline characteristics.

### 3.2. Multivariable ITPW Analysis

Weighted binary logistic regression analysis results for positive margins found that NHB patients were 12% more likely than NHW patients to have a positive margin after resection (OR 1.12, 95% CI 1.01–1.23, *p* = 0.029). Patients treated in the northeastern United States were 9% less likely to have positive margins compared to those treated in the south (OR 0.91, 95% CI 0.84–0.99, *p* = 0.023). In addition, patients treated at academic/research cancer programs were 11% less likely to have positive margins compared to those treated at community cancer programs (OR 0.89, 95% CI 0.83–0.95, *p* = 0.0006). Table 2 summarizes these findings.

### 3.3. Regional Variations in Positive Margins

Within the west, NHB patients were 46% more likely to have positive margins compared to NHW patients (OR: 1.46, 95% CI 1.03–2.09, *p* = 0.036). Similarly, NHB patients operated on in the west were 54% more likely to have positive margins compared to NHO patients (OR 1.54, 95% CI 1.03–2.29, *p* = 0.035). NHB patients in the northeast were 42% less likely to have positive margins compared to NHB patients operated on in the west (OR 0.58, 95% CI 0.37–0.89, *p* = 0.012). Similarly, NHW patients in the northeast were 11% less likely to have positive margins compared to NHW patients operated on in the west (OR 0.89, 95% CI 0.80–0.99, *p* = 0.037) (Table 3).

### 3.4. Variations in Positive Margins by Type of Facility

Within academic/research programs, NHB patients were 53% more likely than NHO patients to have positive margins (OR 1.53, 95% CI 1.12–2.07, *p* = 0.007). NHW patients treated in community cancer programs were 12% (OR 1.12, 95% CI 1.03–1.20, *p* = 0.007) more likely to have positive margins compared to NHW patients treated in academic/research programs. Similarly, NHW patients treated in integrated network cancer programs were 15% (OR 1.15, 95% CI 1.05–1.26, *p* = 0.002) more likely to have positive margins compared to NHW patients treated in academic/research programs. In addition, NHO patients treated in community cancer programs were 46% (OR 1.46, 95% CI 1.06–2.00, *p* = 0.02) more likely to have positive margins compared to NHO patients treated in academic/research programs (Table 4).

## 4. Discussion

Our study, which examines a large national database representative of more than 1500 Commission on Cancer-accredited facilities, demonstrates in a study cohort of nearly 60,000 LARC patients that NHB patients had higher odds of positive CRMs compared to their NHW counterparts, suggesting the existence of a disparity in the management of NHB patients afflicted with rectal cancer. Although prior studies utilizing the NCDB have also demonstrated an increased likelihood (19% to 29%) [16,17,20,21] of positive CRM in NHB patients undergoing rectal cancer resection compared to NHW patients, our study performed an IPTW analysis, a more robust approach than the adjusted logistic regressions performed by prior studies, which demonstrated that NHB patients are 12% more likely to undergo LARC resection with positive margins compared to NHW, and that this disparity may vary between regions of the country and facility types.

Our results indicate that patients undergoing surgery in the northeast appear to have superior outcomes. Although differences in margin positivity status may be multifactorial, the fact that, relative to the rest of the country, there are more National Cancer Institute (NCI) designated cancer centers in the northeast suggests that “cutting edge cancer treatments” may be more common in the northeast [22]. This is consistent with studies reporting adherence to the National Accreditation Program for Rectal Cancer (NAPRC) standards, which demonstrate that, relative to the northeast (New England), performance measure achievement (defined as negative proximal, distal, and circumferential margins and >12 lymph nodes harvested during resection) was inferior in other regions of the country [23].

Although our study controls for multiple potentially confounding variables such as age, sex, Charlson–Deyo score, pathologic stage, days from diagnosis to surgery, pathologic grade, type of surgery, timing of radiation therapy in relation to surgery, insurance type, high school education, and direct distance from patient’s residence to hospital recorded in the NCDB, the persistence of a relative increased likelihood of a positive margin in NHB compared to NHW patients following rectal cancer resection suggests that factors not reported in the NCDB or controlled for in our study may be potential drivers of this disparity. Such factors may include, but are not limited to, (1) hospital volume; (2) surgeon specialization; (3) patient body mass index (BMI); (4) anatomic pelvic variations; and (5) implicit bias.

Hospital volume, a factor that we did not control for, is known to impact margin status [16] as well as mitigate 5-year overall survival rate differences between NHB and NHW patients [24]. These results would suggest that rectal cancer patients be preferentially operated on at high volume centers (HVC). However, since this may not be currently feasible and yet surgeon-specific variables such as certification in colorectal surgery have been associated with a decreased likelihood of obtaining positive CRM [25], triaging rectal cancer patients preferentially to sub-specialists, may be a viable alternative and should thereby minimize disparity in rectal cancer management.

In addition to treatment-related variables, patient-related variables, such as BMI and pelvic anatomic variation, not routinely captured by the NCDB may also be contributing to the disparity in the surgical management of NHB relative to NHW patients. Given that obesity is more prevalent among NHB compared to any other ethnic group and that it can add complexity to the surgical resection of rectal cancers, it is possible that differences in BMI between NHB and NHW patients may account, in part, for the noted disparity [26,27,28]. Similarly, previous studies have also demonstrated differences in pelvic anatomy by race. For example, white women have a wider pelvic inlet and outlet and shallower anterior–posterior outlet than African American women [29]. Since certain pelvimetric variables may be predictive of poor rectal cancer resection and positive CRM [30,31,32], it is possible that anatomical differences between NHB and NHW patients may also contribute to the observed differences in LARC resection margin status.

It is possible that implicit bias, defined as “a preference for a social group that is both unconscious and automatic” [33], may also contribute to the noted disparity in the surgical management of LARC. According to some studies, implicit bias is prevalent among surgeons [34,35] and may therefore impact overall management. In addition, the fact that the disparity persisted even within academic centers suggests that implicit bias may be differentially impacting the extent of attending supervision and engagement during the surgical management of NHB LARC patients.

We acknowledge several limitations in our study. First, the data used were retrospectively collected and therefore subject to bias on the part of data collectors and causality cannot be inferred. An observational prospective study would be needed to confirm whether causality exists. However, the IPTW multivariable analysis that we employed was able to control for the numerous confounding variables collected by the NCDB and allowed us to compare margin positivity rates among racial/ethnic groups in the NCDB. Our ability to control for other potential confounding demographic factors such as higher education/employment status, and access to routine healthcare was also limited by the data captured by the NCDB. 

In conclusion, our study demonstrates that racial disparities in the management of LARC, defined by resection margin status, vary between regions of the country and facility type, even when multiple potential confounders are controlled for. Insofar as subspecialty training and specialization in rectal cancer surgical management influence resection margin status, efforts to increase subspecialty training as well as adherence to programs aimed at optimizing the multidisciplinary and multimodality management of LARC should help reduce disparities in rectal cancer care. Similarly, since BMI and pelvic anatomy impact margin positivity and differ between individuals of varying race and ethnicity, efforts to increase diversity in clinical trial participation are warranted. Such efforts may provide opportunities for improving the surgical approach in individuals with elevated BMI and challenging pelvic anatomy and thereby improve the overall results of rectal cancer management. Lastly, ongoing efforts to educate healthcare providers on minimizing implicit bias may further reduce disparity in rectal cancer management.

## Figures and Tables

**Table 1:** Characteristics of patients with rectal adenocarcinoma in the National Cancer Database between 2004 and 2019 who had positive or negative margins (N = 58,389).

Characteristic	All (N = 58,389)		Non-Hispanic White (N = 47,130)		Non-Hispanic Black (N = 4902)		Hispanic (N = 3652)		Non-Hispanic Other (N = 2705)		*p*-Value
	Median	IQR	Median	IQR	Median	IQR	Median	IQR	Median	IQR	
Age (years) *	62	19	63	19	60	18	59	19	60	18	<0.0001
Time between diagnosis and definitive surgery (days) *	102	111	100	109	111	116	113	118.5	110	114	<0.0001
^a^ Distance between patient’s address and facility (miles) *	10.9	22	12	24.1	7.4	13.7	7.8	11.7	7.7	11.6	<0.0001
Sex *	**N**	**%**	**N**	**%**	**N**	**%**	**N**	**%**	**N**	**%**	<0.0001
Male	33,713	60.26	27,439	60.49	2634	56.69	2154	63.45	1486	58.34	
Female	22,236	39.74	17,922	39.51	2012	43.31	1241	36.55	1061	41.66	
Charlson–Deyo comorbidity score *											<0.0001
0	42,022	75.11	34,031	75.02	3390	72.97	2604	76.7	1997	78.41	
1	10,385	18.56	8375	18.46	944	20.32	619	18.23	447	17.55	
2	2435	4.35	2053	4.53	206	4.43	113	3.33	63	2.47	
3 or more	1107	1.98	902	1.99	106	2.28	59	1.74	40	1.57	
**Pathologic Stage**											0.074
2	24,465	43.73	19,904	43.88	2037	43.84	1446	42.59	1078	42.32	
3	31,484	56.27	25,457	56.12	2609	56.16	1949	57.41	1469	57.68	
**Pathologic Grade**											0.51
Moderately Differentiated	41,880	74.85	33,984	74.92	3472	74.73	2519	74.2	1905	74.79	
Poorly Differentiated	8531	15.25	6890	15.19	733	15.78	523	15.41	385	15.12	
Undifferentiated	982	1.76	813	1.79	72	1.55	57	1.68	40	1.57	
Well Differentiated	4556	8.14	3674	8.1	369	7.94	296	8.72	217	8.52	
Surgery type *											<0.0001
Proctectomy or Proctocolectomy in continuity with other organs, i.e., pelvic exenteration	1845	3.30	1447	3.19	199	4.28	118	3.48	81	3.18	
Proctectomy, ^b^ NOS	434	0.78	362	0.80	31	0.67	27	0.8	14	0.55	
Proctocolectomy, ^b^ NOS	1465	2.62	1203	2.65	130	2.8	81	2.39	51	2.00	
Pull thru w/sphincter preservation, i.e., coloanal anastomosis	3839	6.86	3129	6.9	318	6.84	230	6.77	162	6.36	
^c^ Total proctectomy	12,296	21.98	10,149	22.37	1051	22.62	638	18.79	458	17.98	
^d^ Wedge or segmental resection; partial proctectomy, ^b^ NOS	36,070	64.47	29,071	64.09	2917	62.79	2301	67.78	1781	69.93	
Sequence of radiation treatment relative to surgery, if performed *											0.033
Intraoperative Radiation	23	0.04	21	0.05	.	.	.	.	.	.	
Intraoperative Radiation + other radiation therapy administered before or after surgery	50	0.09	41	0.09	.	.	.	.	.	.	
No radiation treatments given	16,062	28.71	13,104	28.89	1317	28.35	943	27.78	698	27.4	
Surgery followed by radiation treatment	11,530	20.61	9460	20.85	925	19.91	655	19.29	490	19.24	
Radiation therapy both before and after surgery	240	0.43	197	0.43	21	0.45	12	0.35	10	0.39	
Radiation therapy prior to surgery only	28,044	50.12	22538	49.69	2378	51.18	1781	52.46	1347	52.89	
Facility type *											<0.0001
Academic/Research	17,623	31.5	13,450	29.65	1849	39.8	1297	38.2	1027	40.32	
Integrated Network Cancer Program	11,065	19.78	9083	20.02	958	20.62	548	16.14	476	18.69	
Community Cancer Program	27,261	48.72	22,828	50.33	1839	39.58	1550	45.66	1044	40.99	
Insurance *											<0.0001
Private/Managed care	24,525	43.83	20,175	44.48	1791	38.55	1342	39.53	1217	47.78	
Uninsured	2897	5.18	1918	4.23	395	8.5	411	12.11	173	6.79	
Public	28,527	50.99	23,268	51.3	2460	52.95	1642	48.37	1157	45.43	
^e^ Education *											<0.0001
≥29.0%	11,020	19.7	6781	14.95	1868	40.21	1750	51.55	621	24.38	
20.0–28.9%	14,710	26.29	11,873	26.17	1521	32.74	779	22.95	537	21.08	
14.0–19.9%	15,855	28.34	13,890	30.62	803	17.28	513	15.11	649	25.48	
<14.0%	14,364	25.67	12,817	28.26	454	9.77	353	10.4	740	29.05	
^f^ Region of USA *											<0.0001
Northeast	10,749	19.21	9039	19.93	716	15.41	528	15.55	466	18.3	
Midwest	14,782	26.42	13,269	29.25	911	19.61	291	8.57	311	12.21	
South	21,052	37.63	16,303	35.94	2770	59.62	1420	41.83	559	21.95	
West	9366	16.74	6750	14.88	249	5.36	1156	34.05	1211	47.55	

^a^ As calculated by the Haversine formula. ^b^ Not otherwise specified. ^c^ Includes but is not limited to abdominoperineal resection (Miles procedure). ^d^ Includes but is not limited to anterior resection, Hartmann’s operation, low anterior resection, transsacral rectosigmoidectomy, and total mesorectal excision. ^e^ As defined as the proportion of adults in the patient’s ZIP code who did not graduate from high school. ^f^ See Supplement A for states and territories included in each region. * Statistically significant values.

**Table 2:** Inverse probability of treatment weighted (IPTW) ^a^ estimates of odds ratios for positive margins.

Odds Ratio Estimates and Wald Confidence Intervals		95% Confidence Limits		*p*-Value
Description of Characteristics	^b^ OR	^c^ LCL	^d^ UCL	*p*-Value
Race and Ethnicity				
Hispanic vs. Non-Hispanic White	0.96	0.85	1.08	0.45
Non-Hispanic Black vs. Non-Hispanic White	1.12 *	1.01	1.23	0.029
Non-Hispanic Other vs. Non-Hispanic White	0.92	0.80	1.06	0.26
Facility Type				
Academic/Research vs. Community Cancer Program	0.89 *	0.83	0.95	0.0006
Facility Integrated Network Cancer vs. Community Cancer Program	1.03	0.95	1.11	0.48
Region				
Midwest vs. South	0.99	0.92	1.06	0.76
Northeast vs. South	0.91 *	0.84	0.99	0.023
West vs. South	1.05	0.97	1.14	0.24

^a^ IPTW model included age, sex, Charlson–Deyo score, pathologic stage, time from initial diagnosis to surgery, pathologic grade, surgery type, radiation and surgery sequence, insurance status, education status. ^b^ Odds ratio. ^c^ Lower control limit. ^d^ Upper control limit. * Statistically significant values.

**Table 3:** Significant differences in margin positivity rates between race/ethnicity across regions of the United States.

Race/Ethnicity	In Region	Compared to Race/Ethnicity	In Region	^a^ OR	^b^ LCL for OR	^c^ UCL for OR	*p*-Value
Non-Hispanic Black	West	Non-Hispanic White	West	1.46 *	1.03	2.09	0.036
Non-Hispanic Black	West	Non-Hispanic Other	West	1.54 *	1.03	2.29	0.035
Non-Hispanic Black	Northeast	Non-Hispanic Black	West	0.58 *	0.37	0.89	0.012
Non-Hispanic White	Northeast	Non-Hispanic White	West	0.89 *	0.80	0.99	0.037

^a^ Odds ratio. ^b^ Lower confidence limit. ^c^ Upper confidence limit. * Statistically significant values.

**Table 4:** Significant differences in margin positivity rates between race/ethnicity across facility type.

Race/Ethnicity	In Facility Type	Compared to Race/Ethnicity	In Facility Type	^a^ OR	^b^ LCL for OR	^c^ UCL for OR	*p*-Value
Non-Hispanic Black	Academic/Research	Non-Hispanic Other	Academic/ Research	1.53 *	1.12	2.07	0.007
Non-Hispanic White	Community Cancer Program	Non-Hispanic White	Academic/ Research	1.12 *	1.03	1.20	0.007
Non-Hispanic White	Integrated Network Cancer Program	Non-Hispanic White	Academic/ Research	1.15 *	1.05	1.26	0.002
Non-Hispanic other	Community Cancer Program	Non-Hispanic Other	Academic/ Research	1.46 *	1.06	2.00	0.02

^a^ Odds ratio. ^b^ Lower confidence limit. ^c^ Upper confidence limit. * Statistically significant values.

## Data Availability

Restrictions apply to the availability of these data. Data were obtained from the American College of Surgeons (ACS) National Cancer Database (NCDB) and are available with the permission of ACS.

## Supplementary Materials

The following supporting information can be downloaded at: https://www.mdpi.com/article/10.3390/curroncol31070280/s1, Supplement A. Breakdown of states assigned to each region of the United States of America

## References

[1] Siegel R.L., Miller K.D., Fuchs H.E., Jemal A. (2022). Cancer statistics, 2022. CA Cancer J. Clin..

[2] Sharma I., Kim S., Sridhar S., Basha R. (2020). Colorectal Cancer: An Emphasis on Factors Influencing Racial/Ethnic Disparities. Crit. Rev. Oncog..

[3] Howard R., Hendren S., Patel M., Gunaseelan V.M., Wixson M., Waljee J., Englesbe M., Bicket M.C. (2023). Racial and Ethnic Differences in Elective Versus Emergency Surgery for Colorectal Cancer. Ann. Surg..

[4] May F.P., Almario C.V., Ponce N., Spiegel B.M.R. (2015). Racial Minorities Are More Likely Than Whites to Report Lack of Provider Recommendation for Colon Cancer Screening. Am. J. Gastroenterol..

[5] Snyder R.A., Hu C.Y., Zafar S.N., Francescatti A., Chang G.J. (2021). Racial Disparities in Recurrence and Overall Survival in Patients with Locoregional Colorectal Cancer. J. Natl. Cancer Inst..

[6] American Cancer Society (2023). Cancer Facts & Figures 2023.

[7] Quirke P., Dixon M.F., Durdey P., Williams N.S. (1986). Local recurrence of rectal adenocarcinoma due to inadequate surgical resection. Histopathological study of lateral tumour spread and surgical excision. Lancet.

[8] Mukkai Krishnamurty D., Wise P.E. (2016). Importance of surgical margins in rectal cancer. J. Surg. Oncol..

[9] MERCURY Study Group (2006). Diagnostic accuracy of preoperative magnetic resonance imaging in predicting curative resection of rectal cancer: Prospective observational study. BMJ.

[10] Rickles A.S., Dietz D.W., Chang G.J., Wexner S.D., Berho M.E., Remzi F.H., Greene F.L., Fleshman J.W., Abbas M.A., Peters W. (2015). High Rate of Positive Circumferential Resection Margins Following Rectal Cancer Surgery: A Call to Action. Ann. Surg..

[11] Nagtegaal I.D., Quirke P. (2008). What Is the Role for the Circumferential Margin in the Modern Treatment of Rectal Cancer?. J. Clin. Oncol..

[12] Trakarnsanga A., Gonen M., Shia J., Goodman K.A., Nash G.M., Temple L.K., Guillem J.G., Paty P.B., Garcia-Aguilar J., Weiser M.R. (2013). What is the Significance of the Circumferential Margin in Locally Advanced Rectal Cancer After Neoadjuvant Chemoradiotherapy?. Ann. Surg. Oncol..

[13] Detering R., Rutgers M.L.W., Bemelman W.A., Hompes R., Tanis P.J. (2021). Prognostic importance of circumferential resection margin in the era of evolving surgical and multidisciplinary treatment of rectal cancer: A systematic review and meta-analysis. Surgery.

[14] Lee D.Y., Teng A., Pedersen R.C., Tavangari F.R., Attaluri V., McLemore E.C., Stern S.L., Bilchik A.J., Goldfarb M.R. (2017). Racial and Socioeconomic Treatment Disparities in Adolescents and Young Adults with Stage II–III Rectal Cancer. Ann. Surg. Oncol..

[15] Bocca G., Mastoridis S., Yeung T., James D.R.C., Cunningham C. (2022). Visceral-to-subcutaneous fat ratio exhibits strongest association with early post-operative outcomes in patients undergoing surgery for advanced rectal cancer. Int. J. Color. Dis..

[16] Russell M.C., You Y.N., Hu C.-Y., Cormier J.N., Feig B.W., Skibber J.M., Rodriguez-Bigas M.A., Nelson H., Chang G.J. (2013). A Novel Risk-Adjusted Nomogram for Rectal Cancer Surgery Outcomes. JAMA Surg..

[17] Bakkila B.F., Kerekes D., Nunez-Smith M., Billingsley K.G., Ahuja N., Wang K., Oladele C., Johnson C.H., Khan S.A. (2022). Evaluation of Racial Disparities in Quality of Care for Patients with Gastrointestinal Tract Cancer Treated with Surgery. JAMA Netw. Open.

[18] Chesnaye N.C., Stel V.S., Tripepi G., Dekker F.W., Fu E.L., Zoccali C., Jager K.J. (2022). An introduction to inverse probability of treatment weighting in observational research. Clin. Kidney J..

[19] Deyo R., Cherkin D.C., Ciol M.A. (1992). Adapting a clinical comorbidity index for use with ICD-9-CM administrative databases. J. Clin. Epidemiol..

[20] Naffouje S.A., Ali M.A., Kamarajah S.K., White B., Salti G.I., Dahdaleh F. (2022). Assessment of Textbook Oncologic Outcomes Following Proctectomy for Rectal Cancer. J. Gastrointest. Surg..

[21] Simon H.L., De Paula T.R., Profeta Da Luz M.M., Kiran R.P., Keller D.S. (2021). Predictors of Positive Circumferential Resection Margin in Rectal Cancer: A Current Audit of the National Cancer Database. Dis. Colon Rectum..

[22] Alimena S., Davis M., Pelletier A., Terry K., King M., Feldman S. (2022). Regional Variation in Access to Oncologic Care and Racial Disparities Among Cervical Cancer Patients. Am. J. Clin. Oncol..

[23] Brady J.T., Xu Z., Scarberry K.B., Saad A., Fleming F.J., Remzi F.H., Wexner S.D., Winchester D.P., Monson J.R., Lee L. (2018). Evaluating the Current Status of Rectal Cancer Care in the US: Where We Stand at the Start of the Commission on Cancer’s National Accreditation Program for Rectal Cancer. J. Am. Coll. Surg..

[24] Shannon A.B., Straker R.J., Keele L., Kelz R.R., Fraker D.L., Roses R.E., Miura J.T., Karakousis G.C. (2022). The impact of hospital volume on racial disparities in resected rectal cancer. J. Surg. Oncol..

[25] Justiniano C.F., Aquina C.T., Fleming F.J., Xu Z., Boscoe F.P., Schymura M.J., Temple L.K., Becerra A.Z. (2019). Hospital and surgeon variation in positive circumferential resection margin among rectal cancer patients. Am. J. Surg..

[26] Chern H., Chou J., Donkor C., Shia J., Guillem J.G., Nash G.M., Paty P.B., Temple L.K., Wong D.W., Weiser M.R. (2010). Effects of Obesity in Rectal Cancer Surgery. J. Am. Coll. Surg..

[27] Yuval J.B., Patil S., Gangai N., Omer D.M., Akselrod D.G., Fung A., Harmath C.B., Kampalath R., Krehbiel K., Lee S. (2023). MRI assessment of rectal cancer response to neoadjuvant therapy: A multireader study. Eur. Radiol..

[28] (2022). Centers for Disease Control and Prevention. https://www.cdc.gov/obesity/php/data-research/adult-obesity-facts.html.

[29] Handa V.L., Lockhart M.E., Fielding J.R., Bradley C.S.M., Brubaker L., Cundiff G.W., Ye W., Richter H.E. (2008). Racial Differences in Pelvic Anatomy by Magnetic Resonance Imaging. Obstet. Gynecol..

[30] Chau J., Solomon J., Liberman A.S., Charlebois P., Stein B., Lee L. (2020). Pelvic dimensions on preoperative imaging can identify poor-quality resections after laparoscopic low anterior resection for mid- and low rectal cancer. Surg. Endosc..

[31] Baik S.H., Kim N.K., Lee K.Y., Sohn S.K., Cho C.H., Kim M.J., Kim H., Shinn R.K. (2008). Factors Influencing Pathologic Results after Total Mesorectal Excision for Rectal Cancer: Analysis of Consecutive 100 Cases. Ann. Surg. Oncol..

[32] Boyle K.M., Petty D., Chalmers A.G., Quirke P., Cairns A., Finan P.J., Sagar P.M., Burke D. (2005). MRI assessment of the bony pelvis may help predict resectability of rectal cancer. Colorectal Dis..

[33] Greenwald A.G., Banaji M.R. (1995). Implicit social cognition: Attitudes, self-esteem, and stereotypes. Psychol. Rev..

[34] Zebib L., Strong B., Moore G., Ruiz G., Rattan R., Zakrison T.L. (2019). Association of Racial and Socioeconomic Diversity With Implicit Bias in Acute Care Surgery. JAMA Surg..

[35] Haider A.H., Schneider E.B., Sriram N., Dossick D.S., Scott V.K., Swoboda S.M., Losonczy L., Haut E.R., Efron D.T., Pronovost P.J. (2014). Unconscious race and class bias: Its association with decision making by trauma and acute care surgeons. J. Trauma Acute Care Surg..

