# Supplementary material for: Disparities in Outcomes following Resection of Locally Advanced Rectal Cancer
Source: Curr Oncol. 2024 Jun 30;31(7):3798–807. doi: 10.3390/curroncol31070280 (PMC11275254; doi:10.3390/curroncol31070280)
Supplement: Supplementary file 1 [file curroncol-31-00280-s001.zip › curroncol-3078302-supplementary.pdf]

Supplement: Breakdown of states assigned to each region of the United States of America

Northeast: Connecticut, Massachusetts, Maine, New Hampshire, Rhode Island, Vermont, New Jersey, New York, Pennsylvania

South: Washington, D.C., Delaware, Florida, Georgia, Maryland, North Carolina, South Carolina, Virginia, West Virginia, Alabama, Kentucky, Mississippi, Tennessee, Arkansas, Louisiana, Oklahoma, Texas

Midwest: Illinois, Indiana, Michigan, Ohio, Wisconsin, Iowa, Kansas, Minnesota, Missouri, North Dakota, South Dakota

West: Arizona, Colorado, Idaho, Montana, New Mexico, Nevada, Utah, Wyoming, Alaska, California, Hawaii, Oregon, Washington
